# Can Cranioplasty Be Considered a Tool to Improve Cognitive Recovery Following Traumatic Brain Injury? A 5-Years Retrospective Study

**DOI:** 10.3390/jcm10225437

**Published:** 2021-11-21

**Authors:** Francesco Corallo, Viviana Lo Buono, Rocco Salvatore Calabrò, Maria Cristina De Cola

**Affiliations:** IRCCS Centro Neurolesi “Bonino-Pulejo”, Via Palermo S.S. 113, C.da Casazza, 98124 Messina, Italy; francesco.corallo@irccsme.it (F.C.); roccos.calabro@irccsme.it (R.S.C.); mariacristina.decola@irccsme.it (M.C.D.C.)

**Keywords:** cranioplasty, cognitive improvement, traumatic brain injury, neuropsychology

## Abstract

Cranioplasty (CP) is a neurosurgical intervention of skull repairing following a decompressive craniectomy. Unfortunately, the impact of cranioplasty on cognitive and motor function is still controversial. Fifteen TBI subjects aged 26–54 years with CP after decompressive craniectomy were selected in this observational retrospective study. As per routine clinical practice, a neuropsychological evaluation carried out immediately before the cranioplasty (Pre CP) and one month after the cranioplasty (T0) was used to measure changes due to CP surgery. This assessment was performed each year for 5 years after discharge in order to investigate long-term cognitive changes (T1-T5). Before cranioplasty, about 53.3% of subjects presented a mild to severe cognitive impairment and about 40.0% a normal cognition. After CP, we found a significant improvement in all neuropsychological test scores. The more significant differences in cognitive recovery were detected after four years from CP. Notably, we found significant differences between T4 and T0-T1, as well as between T5 and T0-T1-T2 in all battery tests. This retrospective study further suggests the importance of CP in the complex management of patients with TBI showing how these patients might improve their cognitive function over a long period after the surgical procedure.

## 1. Introduction

Cranioplasty (CP) is a neurosurgical intervention of skull repairing and represents a second-line procedure in patients who have undergone decompressive craniectomy (DC) following a traumatic brain injury (TBI) [1], middle cerebral artery (MCA) infarction, or removal of cranial vault tumors [2]. The most appropriate time to perform cranioplasty, as well as its effect on functional outcome, remains debatable. Indeed, multiple confounding factors, including the material used, surgical technique, cognitive function, and general medical complications, seem to affect early and long-term outcomes [3,4,5].

In particular, the impact of cranioplasty on cognitive and motor function is also controversial. Patients with TBI show a wide range of neurocognitive and psychologic deficits after DC [6]. Although several studies have documented clinical improvements after cranioplasty in patients with severe brain injury, the reasons behind the possible mechanisms that induce such clinical improvement are not fully understood [7]. Patient improvement could be due to reduction in local cerebral compression caused by atmospheric pressure and increased cerebrospinal fluid hydrodynamics with potential improvement in local and global cerebral hemodynamics, blood flow, and metabolism [8].

A neuropsychological assessment is the best approach for understanding the nature, the severity, and the modality of cognitive complaints. This important measure of outcome is much more representative of the prognosis of neurosurgical patients than other outcome scales [9]. When cognitive complaints are reported or persist following brain injury, neuropsychological testing is useful for addressing diagnostic issues as well as treatment and rehabilitation planning [10].

This is why, beyond motor improvements, neurological rehabilitation should also be focused on cognitive functions recovery after a CP, especially during the first months after the surgery procedure [11,12]. However, only a limited number of studies on long-term neurological outcomes after a CP surgery are available, and very few concerning the cognitive recovery of these patients [13,14].

The purpose of this study was then to observe the long-term effects on cognitive recovery of patients with TBI after cranioplasty.

## 2. Materials and Methods

Twenty-two subjects with TBI submitted to decompressive craniectomy and attended the Neuro-rehabilitation Unit of the IRCCS Centro Neurolesi “Bonino Pulejo” of Messina between January 2015 and December 2020. However, five patients were excluded because of missing data, and two died due to unspecified causes. Therefore, fifteen subjects (five women and ten men), aged 26–54 years, were selected and included in this retrospective study. Data was extracted from the hospital database.

As per routine clinical practice, a neuropsychological evaluation carried out immediately before the cranioplasty (Pre CP) and 1 month after the cranioplasty (T0) was used to measure changes due to cranioplasty surgery. This assessment was performed each year for 5 years after discharge in order to investigate long-term cognitive changes (T1–T5).

A standardized battery of tests was used to measure in detail the main cognitive areas involved in TBI. The assessment included a test to globally evaluate cognitive functions, i.e., the Mini-Mental Status Examination (MMSE) [15], and specific scales to investigate multiple cognitive domains, such as memory (the Rey Auditory Verbal Learning Test [16] -RAVLI immediate and RAVLR recall- and Digit Span [17]), comprehension (Token Test [18]) and executive functions and attention (Trail Making Test-TMT [19]). In addition, the Hamilton Rating Scale for depression (HAM-D) and anxiety (HAM-A) [20,21] were also administered to evaluate the possible impact of mood and anxiety on cognition. For all scales and tests, the Italian language validated versions were used. A complete description of these assessments is provided in Table 1.

In order to avoid the ‘practice or learning effects’ related to the repeated experience with the task, similar tests (but with different items for the same task) were administered, when possible.

### Statistical Analysis

The Lilliefors (Kolmogorov–Smirnov) test was used to verify variables’ normality, whereas the Levene test to assess the equality of variances among times. Because of reduced sample dimension, the not-normality of all variables, and the homoscedasticity of almost all of them, we chose a no-parametrical approach to perform inferential statistical analysis. Thus, the Wilcoxon signed-rank test was applied to detect significant pre-post cranioplasty changes of neuropsychological outcomes, and the Friedman test was used to compare these outcomes at different time points (T0–T5) in order to assess changes over time after the CP. On the variables in which the Friedman test detected a significance, the Conover test was applied considering the Bonferroni’s correction (post-hoc analysis). All analysis was performed by using the 4.0.5 version of the open-source software R, by setting *p* < 0.05 as the significance level.

## 3. Results

Table 2 and Table 3 report a detailed description of each subject before and after CP, respectively. Through MMSE cut-offs, we subdivided the sample in patients with nearly normal cognition, mild, and/or severe cognitive impairment. Before CP, about 53.3% of subjects presented a mild to severe cognitive impairment, about 40.0% a normal cognition, and in one subject, the MMSE was not administrable. Notably, in 2 of the 15 subjects, the tools concerning attention, executive functions, and memory before CP (Table 2), as well as at one month-follow-up, were not administrable (Table 3). However, over two years (i.e., at T2), all of our patients were able to complete the assessment. In addition, after CP, we found a significant improvement in all neuropsychological test scores (Table 3).

**Figure 1:**
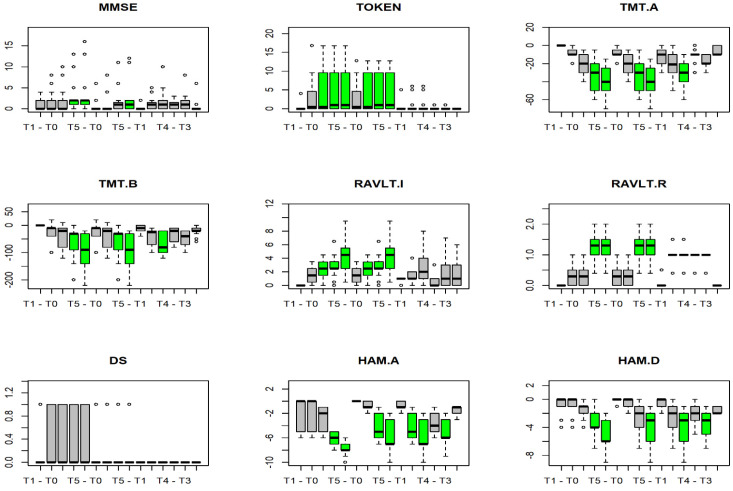
Boxplot of differences between times (T) for each neurophysiological test. T indicates the different time points.

Friedman’s test detected significant differences in all outcomes except Digit Span, as reported in Table 4. All significant differences found by the post-hoc test are depicted in green in Figure 1.

Despite the absence of significant changes in Digit Span scores, the RAVLT test scores showed a gradual improvement in memory occurring over the years. The more significant differences in cognitive recovery have been detected after four years from cranioplasty. Indeed, no significant changes between T0 and T1, as well as between T1 and T2 emerged. On the contrary, we found significant differences between T4 and T0-T1, as well as between T5 and T0-T1-T2 in all battery tests. In addition, significant differences between T3 and T1 also emerged in TOKEN scores (*p* = 0.003).

## 4. Discussion

Cranioplasty following brain surgery is still a thorny debate. Several studies focusing on the materials used, surgical techniques, and timing to perform CP are present in the literature. However, a recent retrospective study [22] on a cohort of 40 patients with DC following severe TBI, compared with a reference population of 115 patients with DC due to other conditions, reports that a successful cranioplasty predicts a favorable outcome after 1 year, whereas patient outcome as assessed before cranioplasty does not predict cranioplasty success or failure.

To the best of our knowledge, this is the first study dealing with long-term cognitive and emotional outcomes of patients with TBI who underwent decompressive craniectomy. The findings of this retrospective study showed that cognitive performance may continue to improve over the years after cranioplasty, and in some cases, until a nearly complete neurological recovery. Overall, the more significant differences in cognitive recovery in our sample have been detected after four years from CP, given that no significant changes between T0 and T1, as well as between T1 and T2 emerged.

The improvement in neurological status after CP was not so surprising for different reasons. First, we must consider that our patients were young, hence with a higher “cognitive reserve” due to a better neuronal plasticity; and that patients following TBI usually present a better recovery, even after months/years from the acute event [23]. The mechanisms subtending these improvements after CP could be the increase of cerebral blood flow and neural metabolism, as well as changes in cerebrospinal fluid hydrodynamic. In order to repair structural defects caused by DC, cranioplasty seems to be the best way to balance the atmospheric pressure on the cranial defects. Increasing the overall intracranial compliance, cerebrospinal fluid velocity, and the flow in the craniospinal junction may promote better blood flow circulation, especially at the cerebral cortex level, also the modifying metabolic gap. Therefore, the increase in cerebral blood flow could be the key component to boost neuralplasticity and then motor and cognitive functioning [5,12]. After all, cranioplasty can remarkably improve cerebrospinal fluid (CSF) dynamics and provide cortical perfusion for both the ipsilateral and contralateral hemispheres. In their study, Shahid et al. [24] reported an improvement in cerebral perfusion in different lobes in around 94% of patients. Sarubbo et al. [25] observed a progressive decline of cortical perfusion in the injured hemisphere and a stable perfusion in the contralateral hemisphere after surgery, hypothesizing a possible role of cranioplasty in restoring flow to meet the prevailing metabolic demand. An increase in CSF flow and CBF with a potentially related improvement in cognitive function was also observed by other authors. The causative impact of CSF on neurological function, however, requires further study.

Second, patients performed neuropsychological rehabilitation during the hospitalization, which promoted an improvement in cognitive function [26,27,28]. Therefore, part of the recovery could be due to such specific training. Previous studies have shown that if an intensive and multidisciplinary rehabilitation program starts early, then the cognitive and motor recovery will be better. Therefore, it is possible to attribute part of the recovery occurring immediately after the CP to this surgical procedure, while the gradual improvement of cognitive and functional outcomes is probably due to a competitive effect between the surgical procedure and rehabilitation [29,30,31].

Third, most of the subjects did not have a severe cognitive impairment before CP. It is well-known that the severity of the brain injury and the degree of pre-cranioplasty deficits may influence the degree of cerebral blood flow improvement after CP and the patient’s subsequent neurological recovery [22]. However, in our study, in three subjects with a severe deficit in attention, executive functions, and memory before CP, and/or at one-month follow-up, the improvement was so high over three to four years after the injury that they were eventually able to complete the assessment with satisfactory scores.

Only a few studies have strictly focused on cognitive recovery after this surgical procedure, and the assessment was often performed with different tools [4,5,6,7,8]. On the contrary, many studies have aimed to understand the right timing to perform cranioplasty after DC. Thus, most studies report about functional and cognitive improvements in patients with severe TBI, especially when the surgical procedure has been performed within three to twelve months after the event [23]. Indeed, early CP was found to improve cognitive function by restoring CSF hydrodynamics, intracranial compliance, and cerebral blood flow when neurocognitive changes are at their peak [26]. However, when is the right timing to perform CP is still controversial because it variably affects functional recovery and is also a risk factor for infections and other complications [32,33]. Archavlis et al. [34], in a 10-year retrospective study on a cohort of 200 patients, observed that patients who performed CP within 7 weeks from the decompressive craniectomy had an improvement of 78% (measured by the GCS) versus the 46% observed in patients who underwent cranioplasty after 7–12 weeks and only 12% after 12 weeks. Di Stefano et al. [35] found a low probability of complications when CP was performed within 3 months from the decompressive craniectomy, whereas the work by Corallo et al. [36] sustains that the timing of cranioplasty is independent of neurologic outcomes. However, the recent review by De Cola et al. [12] concluded that CP performed within 3 months from DC may lead to greater effects on motor functions, whereas for the cognitive domain, the best choice seems to be performing CP from 3 to 6 months, especially if the patient has received neuropsychological rehabilitation.

The novelty of this study is the long-term observation of cognitive and emotional outcomes in the TBI population after cranioplasty. In our opinion, long-term cognitive follow-ups of patients are fundamental to understand if there was a complete recovery, as the significantly improvement over the years highlighted by our findings. Beyond the recovery immediately observed after cranioplasty, this study reports a clear improvement even in patients who initially had a minor and slow recovery. In this prospective, the timing of cranioplasty could become less important, since the effects can distribute over time, and patients continued to improve.

Last but not least, our findings also report a significant improvement in mood, most likely concurring with long-term acceptance of the traumatic event and the return to a normal life.

The main limitation of the study consists in the small sample size, and therefore our results, though promising, needs to be interpreted with caution. Larger and multicenter studies should be fostered to confirm these promising findings. Moreover, the retrospective nature of the study may cause some information bias since we used existing records, as well as a bias due to the lack of a control group. However, we did not assess CP effects on recovery, but we only observed the recovery over time. In fact, the long-term follow-ups and the complete neuropsychological battery represent the main strength of the study.

## 5. Conclusions

This retrospective study further suggests the importance of CP in the complex management of patients with TBI showing that patients might continue to improve their cognitive function over a long period after the surgical procedure. Further larger sample prospective studies with longer follow-up period are needed to confirm these findings and better clarify the role of CP and neuropsychological rehabilitation in the functional recovery of these frail patients.

## Figures and Tables

**Table 1:** The neuropsychological battery used for the assessment.

Neuropsychological Assessment
Mini Mental State Examination (MMSE) The MMSE is a 30-point scale commonly used by healthcare providers to evaluate the global cognitive state as a screening test. The time of administration is about 10 min. Each correct answer provides 1 point. A score < 24 indicates cognitive impairment.
Rey Auditory Verbal Learning Test (RAVLT) This test is divided into two parts: Immediate (RAVLT.I) and Delayed Recall (RAVLT.R). In the Immediate Recall, the examiner reads 15 words and asks the patient to repeat all the memorized words in the patient’s preferred order. This task provides information about episodic verbal memory, encoding and learning strategies. For the evaluation of the long-term memory (RAVLT.R), the patient is asked to repeat the memorized words from the same 15-words list, after 15 min. In the meantime, between the Immediate and the Delayed Recall tests, a nonverbal and visuospatial test was administered in order to avoid any interference with the memory processes. Cut-off: 28.53 (RAVLT.I) and 4.69 (RAVLT.R)
Digit Span (DS) The DS test is a widely used neuropsychological measure known as a test of attention and working memory. The DS consists of a forward recall part and backward recall part for digit sequences. Each part is considered to assess somewhat different cognitive processes. Cut-off: 3.75
Token The Token test is used as a selective measure for the presence of aphasia and as an indicator for the severity of aphasia. All commands in the test consist of no redundant words, referring to circles and rectangles in different colors and sizes (large and small). To perform the requested action, every content word has to be decoded. Cut-off: 26.50
Trail Making Test (TMT) The Trail Making Test is a widely used test to assess executive function in patients with neurological disease. Successful performance of the TMT requires a variety of mental abilities, including letter and number recognition mental flexibility, visual scanning, and motor function. The task requires connecting 25 circles distributed over a sheet of paper. In Part A, the circles are numbered 1–25, and the patient should draw lines to connect the numbers in ascending order. In Part B, the circles include both numbers and letters; here, the patient draws lines to connect the circles in an ascending pattern but alternating between the numbers and letters (i.e., 1-A-2-B-3-C, etc.). Trails are traced in the shortest time possible and without lifting the pen from the paper. Cut-off: 93 (A) and 282 (B)
Hamilton Rating Scale for Depression (HAM-D) The HAM-D is the most widely used clinician-administered depression assessment scale. The 24-item version includes 24 items scored either on a 3-point or 5-point Likert-type scale (i.e., 10 items are defined from 0 to 2, and 14 items are defined from 0 to 4). A score ≥ 8 points defines depression as follows: a score ranged from 8–19 points defines a mild depression, a score ranged from 20–34 points defines a moderate depression, and a score ≥ 35 points defines a severe depression. Cut-off: 7
Hamilton Rating Scale for Anxiety (HAM-A) The scale consists of 14 items, each defined by a series of symptoms, and measures both psychic anxiety (mental agitation and psychological distress) and somatic anxiety (physical complaints related to anxiety). Each item is scored on a scale of 0 (not present) to 4 (severe), with a total score ranged from 0–56, where below 17 indicates mild severity, 18–24 mild to moderate severity, and 25–30 moderate to severe. Cut-off: 17

**Table 2:** Sample characteristics before reconstructive surgery.

ID	Sex	Age ^1^	MMSE	TMT.A	TMT.B	RAVLT.I	RAVLT.R	TOKEN	DS	HAM.A	HAM.D
01	M	26	26	200	346	28.53	4.6	30	6	18	21
02	M	46	8	NA	NA	NA	NA	24.5	NA	16	18
03	F	41	24	280	512	31.25	3.3	33.5	5	19	21
04	M	29	NA	NA	NA	NA	NA	NA	NA	NA	NA
05	M	33	23	350	600	26.5	3.6	32.7	4	20	21
06	M	46	21	206	585	21.5	1.5	9.5	2	18	19
07	F	54	23	350	600	26.5	3.6	32.7	4	20	21
08	M	36	21	206	585	21.5	1.5	9.5	2	18	19
09	M	35	18	200	458	26	5	14.5	4	14	16
10	F	34	25	289	562	32.25	4.3	32.5	6	16	18
11	M	28	26	200	346	28.53	4.6	30	6	18	21
12	M	45	21	206	585	21.5	1.5	9.5	2	18	19
13	M	37	22	206	585	21.5	1.5	9.5	2	18	19
14	F	39	25	289	562	32.25	4.3	32.5	6	16	18
15	F	42	24	280	512	31.25	3.3	33.5	5	19	21

^1^ Age is expressed in years. LEGEND: MMSE = Mini Mental State Examination; TMT = Trail Making Test; RAVL.I = Rey Auditory Verbal Learning (immediate); RAVL.R = Rey Auditory Verbal Learning (recall); HAM.D = Hamilton Rating Scale for depression; HAM.A = Hamilton Rating Scale for anxiety; DS = Digit span.

**Table 3:** Sample characteristics one month after reconstructive surgery.

ID	DC-CP ^1^	MMSE	TMT.A	TMT.B	RAVLT.I	RAVLT.R	TOKEN	DS	HAM.A	HAM.D
01	1	28	60	100	29.53	7.6	32	7	12	10
02	9	10	NA	NA	NA	NA	26.5	NA	10	12
03	4	28	81	100	34.5	7	32.5	7	9	6
04	1	7	NA	NA	NA	NA	8.25	NA	NA	NA
05	9	28	55	120	33.5	7.6	32.7	6	10	10
06	10	26	90	240	30.5	6.5	20.4	6	10	6
07	11	28	55	120	33.5	7.6	32.7	6	10	10
08	12	26	100	240	30.5	6.5	20.4	6	10	6
09	12	24	120	300	38	9	30.25	5	10	12
10	8	26	80	180	33.5	6.7	32.5	6	10	10
11	2	28	60	100	29.53	7.6	32	7	12	10
12	11	26	90	240	30.5	6.5	20.4	6	10	6
13	12	26	100	240	30.5	6.5	20.4	6	10	6
14	9	26	80	180	33.5	6.7	32.5	6	10	10
15	6	28	81	100	34.5	7	32.5	7	9	6
Pre-Post changes *p*-values		0.001	0.002	0.002	0.002	0.002	0.014	0.003	<0.001	0.001

^1^ The time passed between compressive craniectomy and cranioplasty (DC-CP) is expressed in months. LEGEND: MMSE = Mini Mental State Examination; TMT = Trail Making Test; RAVL.I = Rey Auditory Verbal Learning (immediate); RAVL.R = Rey Auditory Verbal Learning (recall); HAM.D = Hamilton Rating Scale for depression; HAM.A = Hamilton Rating Scale for anxiety; DS = Digit span.

**Table 4:** Subjects’ scores over time, comparison analysis results by Friedman’s test and Conover test.

Assessment		Scores at Each Examination (Median (First-Third Quartile))					Friedman Test	Post-Hoc Analysis	
	T0	T1	T2	T3	T4	T5	*p*-Value	Sign. Diff.	*p*-Value *
MMSE	26.0 (26.0–28.0)	28.0 (6.0–28.0)	28.0 (26.0–28.0)	28.0 (26.0–28.0)	28.0 (27.0–29.0)	28.0 (27.0–29.5)	<0.001	T4-T0 T5-T0 T5-T1	<0.001 <0.001 0.003
TMT.A TMT.B	81.0 (60.0–90.0) 180.0 (100.0–240.0)	81.0 (70.0–100.0) 180.0 (110.0–240.0)	80.0 (65.0–90.0) 170.0 (105.0–200.0)	60.0 (135.0–165.0) 160.0 (105.0–170.0)	50.0 (45.0–55.0) 100.0 (95.0–150.0)	40.0 (40.0–50.0) 90.0 (80.0–95.0)	<0.001	T4-T0 T5-T0 T4-T1 T5-T1 T5-T2	<0.001 <0.001 <0.001 <0.001 0.001
RAVLT.I RAVLT.R	33.5 (30.5–33.5) 7.0 (6.5–7.6)	33.5 (30.5–33.5) 7.0 (6.5–7.6)	34.0 (31.2–35.7) 7.0 (7.0–7.6)	35.0 (32.0–36.0) 7.0 (7.0–7.6)	35.0 (32.5–36.5) 8.0 (8.0–8.0)	36.0 (34.0–39.0) 8.0 (8.0–8.0)	<0.001	T4-T0 T5-T0 T4-T1 T5-T1 T5-T2	<0.001 <0.001 <0.001 <0.001 0.002
DIGIT SPAN	6.0 (6.0–7.0)	6.0 (6.0–7.0)	6.0 (6.0–7.0)	6.0 (6.0–7.0)	6.0 (6.0–7.0)	6.0 (6.0–7.0)	0.05	-	-
TOKEN	32.0 (20.4–32.5)	32.0 (20.4–32.5)	32.0 (30.0–32.7)	32.0 (30.0–32.7)	32.0 (30.0–32.7)	32.0 (30.0-–32.7)	<0.001	T3-T0 T4-T0 T5-T0 T3-T1 T4-T1 T5-T1	0.002 <0.001 <0.001 0.003 <0.001 <0.001
HAM.A	10.0 (10.0–10.0)	10.0 (5.2–10.0)	10.0 (5.5–10.0)	9.0 (5.5–9.0)	4.0 (3.5–5.0)	3.0 (2.0–3.0)	<0.001	T4-T0 T5-T0 T4-T1 T5-T1 T4-T2 T5-T2 T5-T3	<0.001 <0.001 0.003 <0.001 0.003 <0.001 0.007
HAM.D	10.0 (6.0–10.0)	8.0 (5.2–10.0)	9.0 (5.5–10.0)	9.0 (5.5–9.0)	5.0 (4.0–6.0)	3.0 (3.0–4.0)	<0.001	T4-T0 T5-T0 T5-T1 T5-T2 T5-T3	<0.001 <0.001 <0.001 <0.001 0.001

LEGEND: MMSE = Mini Mental State Examination; TMT = Trail Making Test; RAVL.I = Rey Auditory Verbal Learning (immediate); RAVL.R = Rey Auditory Verbal Learning (recall); HAM.D = Hamilton Rating Scale for depression; HAM.A = Hamilton Rating Scale for anxiety. * with the Bonferroni’s correction for six comparisons, the corrected level of significance was 0.008.

## Data Availability

The data presented in this study are available on request from the corresponding author. The data are not publicly available due to Hospital policy.
